# Covariates of Knowledge, Attitude, Practice, and Burdens among the Caregivers of Hypertensive Patients

**DOI:** 10.1155/2023/8866231

**Published:** 2023-08-21

**Authors:** Sudipta Deb Nath, Afrin Sultana Chowdhury, Susmita Dey Pinky, Kazi Mahmuda Akter, Nishat Anjum Nourin, Tonmoy Chowdhury, Hossain Ahmed Fahid, K. M. Shailah Sharmin, Md. Mashud Rana, Nazmul Alam, Md. Moinul Ahsan, Adnan Mannan

**Affiliations:** ^1^Department of Genetic Engineering and Biotechnology, University of Dhaka, Dhaka 1000, Bangladesh; ^2^Disease Biology and Molecular Epidemiology (dBme) Research Group, Chattogram, Bangladesh; ^3^Department of Biotechnology and Genetic Engineering, Noakhali Science and Technology University, Noakhali 3841, Bangladesh; ^4^Department of Pharmacology and Toxicology, Indiana University School of Medicine, Indianapolis, IN, USA; ^5^Chittagong Medical College, Chattogram-4203, Bangladesh; ^6^Department of Obstetrics and Gynaecology, Sir Salimullah Medical College Mitford Hospital-1000, Dhaka, Bangladesh; ^7^Rangamati Medical College, Rangamati 4500, Bangladesh; ^8^Mymensingh Medical College, Mymensingh 2200, Bangladesh; ^9^Department of Pharmacology and Therapeutics, Chittagong Medical College, Chattogram 4203, Bangladesh; ^10^Department of Public Health, Asian University for Women, Chattogram 4000, Bangladesh; ^11^Anesthesia and Intensive Care Unit, 250 Bedded General Hospital, Chattogram 4000, Bangladesh; ^12^Department of Genetic Engineering and Biotechnology, University of Chittagong, Chattogram 4331, Bangladesh

## Abstract

Caregivers of hypertensive patients play a significant role in ensuring adequate patient care and lowering the risk of hypertension-relatedcomplications. Caregivers are ideal study subjects for identifying gaps in hypertension management. Our study aimed to assess the knowledge, attitude, and practice (KAP) of hypertensive patients' caregivers, to identify their extent of involvement in patients' care, and to assess their care-related attributes. A descriptive cross-sectional study was conducted from August 2020 to February 2021 in the eight largest tertiary care medical college hospitals and all eight divisions of Bangladesh, with 949 caregivers enrolled. Data were collected using a pretested interviewer-administered questionnaire through snowball sampling and analyzed using a one-way ANOVA, independent-sample *T*-test, and chi-square test. Among the 949 interviewed caregivers, 541 (57.0%) were female, and 479 (50.5%) were aged 18 to 25 years. The percentage scores regarding overall knowledge, attitude, and practice of the caregivers were 54.83 ± 17.95, 47.95 ± 24.05, and 61.26 ± 17.50, respectively. Caregivers' education, history of hypertension, residence, age, relationship with the patient, occupation, and caregiving duration were significantly associated with the KAP scores. In addition, factors such as relationship with the patient, age, educational status, occupation, residence, and caregiving duration/day had significant correlations with all types of burden. Findings of this study suggest the necessity for awareness programs for the caregivers of hypertensive patients to diminish the gap in their KAP and improve their mental and physical health.

## 1. Introduction

Hypertension is one of the most common and challenging public health issues worldwide [1–3], contributing to the mounting global burden of disease and disability [2]. Being a silent killer by damaging organs gradually and permanently, hypertension contributes to 9.4 million of the total cardiovascular annual deaths (17 million) [4, 5]. Although it was formerly considered a disease in developed countries, hypertension significantly affects low- and middle-income ones, with a prevalence rate of 31.5% of the population [6, 7]. Likewise, in 2017, the prevalence of hypertension among Bangladeshi adults was 40.7% [8]. Hypertension is a chronic condition that imposes a colossal economic burden on the family and the health system.

In low- and middle-income countries, to minimize the burden of hypertension that mainly arises from a lack of knowledge and poor self-care practice, regular counselling of caregivers of hypertensive patients can be helpful [9]. A caregiver is defined a relative/friend/neighbour assisting the patient without any compensation [10]. Family members play a pivotal role in making the patient feel safe and supported during the disease period by conveying serenity, courage, and strength [11]. It was also observed that family caregivers devote time and energy to the patients with their activities, financial and nutritional support, and medication [11]. Moreover, they have good knowledge about the patients, so they can provide important information to the physicians and sometimes take vital decisions that may impact the patient's health and management [12, 13].

Knowledge, attitude, and practice (KAP) surveys are the most commonly used technique in health-seeking behavior research [14]. Moreover, a lack of knowledge of the disease risk factors contributes to the rising incidence of that disease [15–17]. By exploring what is known and what is done concerning a healthcare-related objective, a KAP study can have a vast impact on the local community. So far, several studies have been conducted to evaluate patients' KAP toward hypertension in diverse populations worldwide. Though in Bangladesh, a study conducted in 2018 focused on evaluating the KAP of hypertensive patients concerning hypertension [7], as far as we are aware, there has been no research conducted on the KAP of caregivers responsible for hypertensive patients. Considering the close relationship between patients and family caregivers in Bangladesh, we undertook this study to evaluate the levels of KAP of hypertensive patients' caregivers and try to identify related factors.

As many aged hypertensive patients require caregivers for their daily livelihood, the caregivers' quality of life might inversely relate to the caregiving burden. Caregivers' burden is defined as a multidimensional interaction of physical, emotional, and economic hardship experienced by the caregiver during caregiving [18]. Caregiving responsibilities have shown challenging demands that may contribute to monetary burdens, erratic behavior, fluctuating emotions, and time deficiency for social and personal enjoyment with motivation deprivation [18, 19]. This study particularly highlighted the level of burden and efforts in terms of physical, emotional, and economic challenges faced by the caregivers of hypertensive patients in Bangladesh to formally recognize their contribution.

## 2. Methods

### 2.1. Study Design and Site

A descriptive cross-sectional study was conducted from August 2020 to February 2021 in out-patient and in-patient departments at eight government hospitals (Chittagong Medical College Hospital, Mymensingh Medical College Hospital, Sir Salimullah Medical College and Mitford Hospital, Dhaka Medical College Hospital, Khulna Medical College, Sylhet MAG Osmani medical College, Rangamati Medical College Hospital, and Comilla Medical College Hospital) in Bangladesh. These specialized hospitals cover patients from all eight divisions as they manage the maximum patient inflow of the country referred from primary and secondary hospitals.

### 2.2. Inclusion and Exclusion Criteria

The eligibility criteria for the participants were as follows: (1) the person who was identified by the hypertensive patient with systolic blood pressure (BP) ≥ 140 mmHg and/or diastolic BP ≥ 90 mmHg with or without antihypertensive/s or normal BP due to taking antihypertensive/s having the most caregiving interaction, (2) age ≥18 years, and (3) the study considered one caregiver who provided day-to-day services to a hypertensive patient; if more than one caregivers were available, the one who gave the most efforts was interviewed. The exclusion criteria were as follows: (1) respondents moderately/severely suffering from any psychiatric illness, and (2) caregivers of pregnant women with hypertension.

### 2.3. Sample Size and Sampling

The Cochran formula was used to calculate the sample size [20]. The sample size was calculated using the following formula:

(1) $$\begin{array}{c} SS=\frac{\left(z2\times \left(p\left(1-p\right)/e2\right)\right)}{\left(1+\left(z2\times p\left(1-P\right)/e2N\right)\right)}=383\text{,} \end{array}$$

where *N* = population Size, *P* = probability, level of significance (0.05), *e* = margin of error (5%), *z* = *z*-score (95%-1.96), and SS = sample size.

In our study, the collected sample size (*n* = 949) was larger than the calculated sample size because a larger sample size detects outliers that might skew the data and provide a reduced margin of error.

### 2.4. Development and Validation of the Survey Questionnaire

Following an extensive literature search, a face-to-face interviewer-administered questionnaire with 60 questions that included rank order scaling, a Likert-like scale, and both open- and closed-ended questions was prepared in English to evaluate KAP and the burdens of hypertensive patients' caregivers [21]. Later, it was translated into Bangla (the local language) for data collection. The questionnaire was checked and validated by a public health specialist, five cardiologists, and medicine specialists. The study questionnaire was piloted with 40 participants not included in the final study. The final questionnaire was upgraded with 54 questions taken from feedback from the pilot test. Other than baseline information, ten knowledge assessment questions, six attitude-related questions, and ten practice-associated questions were included in the final questionnaire. In addition, the extent of caregiving and caregivers' QoL were also assessed.

### 2.5. Data Collection

In this study, most of our participants were reached out through face-to-face interviews. However, a portion of the participants were interviewed through phone calls because of their unavailability at hospital sites. To ensure data quality and minimize potential bias, all collectors underwent comprehensive training to equip them with the necessary skills and knowledge to conduct interviews effectively and uniformly. The data collection team consisted of three physicians supervising three male and four female data collectors for convenient dealing with the participants of both genders. To avoid the influence of the peers, the interviews were carried out in private. The objectives and procedures of the study were explained to the participants in their native language (Bengali). Local translators assisted both collectors and participants in interpreting the local languages. All participants' information was kept confidential.

### 2.6. Variables and the Method of Verification

The knowledge part consisted of multiple-choice questions with 0–1 and 0–5 scores based on the number of correct responses. Responses to the attitude section had a Likert-like scale of −2 to +2 and +1 to −1 (strongly agree/+2, agree/+1, no idea/0, disagree/−1, and strongly disagree/−2). Caregivers attained 1 point for each correct practice and 0 for each incorrect one. The score latitudes were from 0 to 14, −12 to +12, and 0 to 10 for the knowledge, attitude, and practice portion, accordingly. The knowledge, attitude, and practice scores were transformed into percentages by dividing the total score of each part of each respondent by the maximum score of the identical portion and then multiplied by 100 (Table 2 & 3). The extent of caregiving and QoL of the caregivers were assessed using a 0 to 4 grading scale individually.

### 2.7. Statistical Analysis

The data were analyzed using IBM SPSS v.25. The knowledge, attitude, and practice scores are presented as the mean (±sd). One-way ANOVA test and independent-samples *T*-test were used to analyze any difference in the means of KAP scores between/among variables. Pearson's chi-square test was run to evaluate the difference between the categorical variables. *P* values < 0.05 were considered statistically significant.

## 3. Results

### 3.1. Sociodemographic Characteristics of Hypertensive Patients' Caregivers

Among the 949 interviewed caregivers, 541 (57.0%) were female, and 479 (50.5%) were from 18 to 25 years (Table 1). The majority of the study participants, 796 (83.9%), lived in the same home as the patients. More than half (55.5%, *n* = 527) of the caregivers were son/daughters and 21.5% (*n* = 204) were spouses (Table 1). Of the caregivers, 91.3% (*n* = 866) had at least secondary education. Most of the caregivers were students (33.2%, *n* = 315) and in low-income groups (<10,000, BDT, 32.7%). Urban, semiurban, and rural-residing participants were 61.3%, 21.7%, and 17.0%, respectively. In addition, 512 (54.0%) spent less than one hour per day on caregiving (Table 1).

### 3.2. Overall Scores of the Caregivers

The total level of patients' knowledge was 54.83 ± 17.95, the total level of patients' attitude was 47.95 ± 24.05, and the total level of patients' practice was 61.26 ± 17.50 (Table 2).

### 3.3. Supporting Roles of Caregivers

**Figure 1:**
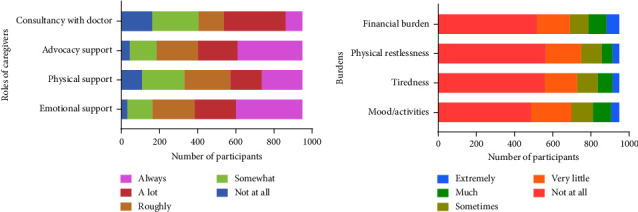
Caregivers' roles and burdens. *X*-axes signify the number of caregivers, and *Y*-axes denote the roles (a) and burdens (b).

Upon asking about the definite role they play regarding caregiving, a scale of 0–4 was implemented, and the scores were 0-not at all, 1-somewhat, 2-fairly, 3-strongly, and 4-always. Most of the participants (>300 respondents in both cases) mentioned that they were always associated (scale 4) with advocacy and providing emotional support to the patient (Figure 1(a)). Rendering physical support was the least demanding role that the caregivers mentioned.

### 3.4. Burdens Associated with Caregiving

While assessing caregivers' perceived level of burden, a scale of 0–4 was applied as an assessment tool. The measurement scale signified the level of burden as 0, implying not at all, 1-very little, 2-somewhat, 3-much, and 4-extreme. In Figure 1(b), most caregivers (above 50%) did not report any financial, physical, or mental burden (scale 0). About 200 caregivers described their facing difficulties as very little (scale 1), whereas only a few caregivers (<100) expressed an extreme level of burden stemming from their liability of caregiving (scale 4).

### 3.5. Factors Affecting KAP

Data analysis revealed that all KAP percentage scores were significantly higher (*P* = 0.004, *a* = 0.002, <0.001) in caregivers living in the same home with hypertensive patients. Caregivers' relationships with the patient were also significantly correlated with their level of knowledge and attitude (*P* < 0.001, <0.001). While the older age had a negative impact on knowledge (*P* < 0.001), the highest score was obtained by the youngest age group (18–26) significantly (Table 3). KAP scores were significantly higher in caregivers with above secondary-level education, *P* value < 0.001, *a* = 0.001, and <0.001, respectively. However, the knowledge and practice scores were significantly better in the group that did not have hypertension. Among the occupations, healthcare professionals scored the highest in knowledge and attitude (*P* < 0.001, <0.001). The inhabitants of urban areas had better KAP towards hypertension than those from rural or suburban areas (*P* < 0.001, <0.001, *a* = 0.001). A significant association was noted between the level of attitude and caregiving duration/day (*P* < 0.001), and it was better for those who spent more than 15 hours with their patients. Significantly higher knowledge was observed amongst the caregivers who were caregiving for ten years or less (*P* = 0.036), but being caregivers for 1–10 years showed better practice (*P* = 0.002). The knowledge level was high for those having >3 hypertensive patients in family (*P* = 0.037), but better practice was noticed among them with 2-3 patients (*P* = 0.013) (Table 3).

### 3.6. Factors Associated with the Burden of Caregiving

Table 4 presents the relationship between caregivers' sociodemographic information and burden (Figure 4). More than 65% of them live always/sometimes with patients confronted with no/little burden during caregiving (*P* = 0.026, 0.049, 0.005). The relationship with the patient was statistically significant correlating with all difficulties (*P* < 0.001, <0.001, <0.001, <0.001). Percentages of not having any tiredness (65.4%) and an economic burden (56.6%) were higher in male caregivers, *P* value = 0.003, *a* = 0.020, respectively. However, the rates of feeling that burdens were high among those aged 26 years or more (*P* value = 0.006, <0.001, = 0.001, <0.001). Those who had a secondary-level education or less faced burdens at a higher level (*P* < 0.001 in all four cases). Percentages of encountering severe trouble among homemakers were exclusively high in all four cases (*P* < 0.001). Another variable showing a significant influence on all burdens was the residence of the caregivers. Besides, 55.1% of the caregivers who spent more than ten years complained of being at a minimum level of tiredness (*P* = 0.001). Significant associations were noted between the burden and spent time/day for caregiving (*P* < 0.001 in every case). Those who are not hypertensive themselves found not/less affected regarding their mood (75.8%) (*P* = 0.003), tiredness (79.1%) (*P* = 0.001), and restlessness (81.4%) (*P* < 0.001) (Table 4).

## 4. Discussion

This study aimed to explore the status of knowledge and mental burden of the caregivers of hypertensive patients in Bangladesh, attributing it to the presence of other sociodemographic correlates. A study from Uzbekistan showed that 64.6% of the primarily diagnosed hypertensive patients had adequate knowledge about hypertension. However, patients acquire more knowledge about their own diseases over time. But the majority of the caregivers in this study also presented fair general knowledge (54.83%) about hypertension [15]. Since more than half of our respondents were from urban areas, this high depth of knowledge about this chronic disease was quite expected. For instance, most respondents could successfully identify hypertension as a risk factor for stroke. Similar results were reported in a study from Sweden where 90% of the patients who already suffered from stroke could mention hypertension as one of the contributing factors [16]. The knowledge score on knowing the names of antihypertensive medicines taken by their patients was higher than the awareness score about the side effects of the medications. This might be explainable since the adverse effects of antihypertensive drugs are generally not that common [22]. Overall, most of the participants had good knowledge about the age group at risk of hypertension, the danger signs of hypertension, and could identify hypertension as a risk factor for stroke. In the current study, age was negatively correlated with knowledge level. The younger participants (age 18–25) scored the highest, whereas the middle-aged caregivers (36–40 years) scored the least. This may be explained because young caregivers are more adept at seeking health knowledge. Moreover, the daughter/son of the caregivers, the highly educated, those who stayed in the same house as the patients, the healthcare professionals, those who lived in urban areas, and those who had more than three hypertensive patients at home showed better knowledge scores than others.

The total attitude score was 47.95 ± 24.05, which was the lowest compared to the knowledge and practice scores. Only a few caregivers showed a positive attitude regarding lifestyle measures that help maintain normal blood pressure. However, the participants were well aware of the importance of avoiding extra salt, which is indeed a good sign, as a high intake of dietary salt is a risk factor for hypertension and other noncommunicable diseases. A study on rural Latino caregivers' revealed a similar finding as the caregivers attempted multiple strategies to limit the consumption of excess salts by their children to reduce the risk of high blood pressure at a young age [22]. A great number of participants supported discontinuing medication when blood pressure remained normal, proving their incognizance of the pathology of hypertension. Moreover, good attitudes were shown by the caregivers when it came to medication adherence during high blood pressure and regular exercise. In Bangladesh, it is commonly seen that there is a wide acceptance of herbal medicine mostly among the rural habitats and to some extent among the urban inhabitants. In this study, some participants thought that herbal medicine could control hypertension.

Regarding the practice, the respondents showed a fair level of it (61.26 ± 17.50). However, those who are hypertensive patients themselves may get exhausted due to their own disease history and a long course of treatment and, therefore, show less compliance. More than half of the caregivers answered that they encourage their patients to exercise regularly, which aligns with the recommendation of 30 minutes of physical activity by the World Health Organization (WHO) [23]. Participants exhibited good practice in being conscious of the danger signs of hypertension, reminding the patients about taking medications correctly, and helping to avoid stress and forbidden foods. Besides, participants who shared the same house with their patients exhibited a higher impact on the practice level.

Our study shows that spouses, daughter/son, and sister/brother carried out responsibilities at a higher percentage. Moreover, a Nigerian study on caregivers of hypertensive patients found that 46% were patients' spouses [24]. Education is found to be a positive factor that affects our participants' KAP levels. This was expected as higher education increases awareness regarding health issues and provides access to the required information. Hence, focusing on arranging educational programs, primarily targeting older populations and rural dwellers may help raise the KAP level. The positive family history of hypertension also showed a significant relationship with the caregivers' knowledge and practice. The higher the number of hypertensive patients in the family, the higher the knowledge level was. The suffering of multiple members of the family may influence them to learn more about the disease. On the contrary, the practice level was the lowest for the caregivers if there had been >3 hypertensive patients in the family, portraying caregivers' exhaustion.

We also asked our participants about how much caregiving stress affects their overall QoL. Homemakers, rural residents, and caregivers with low literacy levels perceived more burden in the process of caregiving. These groups of caregivers also had lower KAP scores. Therefore, it seems that their increased stress might be influenced negatively by their KAP level or vice versa. In addition, this study found that female caregivers were feeling more tired and financially loaded. Another possible contributor to the caregiver's burden is the duration of caregiving. A study conducted on stroke patient's caregivers in Gilan province of Iran showed that the duration of caregiving was <5 years for 84.4% of family caregivers [25]. However, the caregiving period was more extensive in the case of hypertensive patients, which might extend to >10 years as it is a long-term illness. Our study demonstrated the association of 1–5 years of caregiving with a higher percentage of tiredness. This might be associated with new-onset lifestyle changes with reduced sleep time, high levels of stress, and anxiety, leading to the point of an individual's burnout syndrome. Because of the cost and amount of antihypertensive drugs for a long period, it was observed that more than 10 years of caregiving financially burdened the caregivers to the highest extent. Our study also found that caregivers who were hypertensive (51%) faced more exhaustion than the nonhypertensive ones. This can be explained as the additional burden of responsibilities from caring for a chronically ill patient that leads to increased stress and physical, mental, and emotional exhaustion that eventually results in neglecting their own health condition.

## 5. Conclusion

This study showed a fair level of knowledge and fair practice among the caregivers. However, the relatively poor attitude score of the caregivers raises a crucial concern that needs to be addressed. Our data indicate that specific attention is needed if they are less educated, unemployed, hypertensive patients themselves, and caring for patients for a more extended period which can affect their KAP score and stress level, and eventually they may feel vulnerable. So, our findings support the call for specific learning programs for caregivers, to reduce the gap in their knowledge and avoid emergency conditions by improving their awareness and practice. Moreover, further attention to the mental and physical health of the caregivers may help to improve their QoL which will ultimately enhance their caregiving role.

## Figures and Tables

**Table 1:** Baseline sociodemographic characteristics of the caregivers.

Characteristics	Categories	Total *N* *=* 949 number (%)
Age of the caregiver (in years)	18–25	479 (50.5)
	26–30	137 (14.4)
	31–35	83 (8.7)
	36–40	55 (5.8)
	>40	195 (20.5)

Relation with the patient	Spouse	204 (21.5)
	Daughter/son	527 (55.5)
	Daughter-in-law/son-in-law	46 (4.8)
	Sister/brother	31 (3.3)
	Others	141 (14.9)

Sex	Male	408 (43.0)
	Female	541 (57.0

Shared same home	Yes	796 (83.9)
	No	38 (4.0)
	Sometimes	115 (12.1)

Education	Never attended school	17 (1.8)
	Primary	66 (7.0)
	Secondary	113 (11.9)
	Higher secondary	280 (29.5)
	Tertiary	473 (49.8)

Occupation	Student	315 (33.2)
	Homemaker	197 (20.8)
	Service holder	120 (12.6)
	Business	93 (9.8)
	Health professionals	191 (20.10)
	Others	33 (3.5)

Monthly income (taka)	<10,000	310 (32.7)
	10,000–24,999	192 (20.2)
	25,000–49,999	260 (27.4)
	50,000–74,999	99 (10.4)
	75,000–100,000	68 (7.2)
	>100,000	20 (2.1)

Residence	Rural	161 (17.0)
	Semiurban	206 (21.7)
	Urban	582 (61.3)

Duration of being the primary caregiver	<1 year	82 (8.6)
	1–5 years	525 (55.3)
	5–10 years	208 (21.9)
	>10 years	134 (14.1)

Number of hypertensive people in the family	1	498 (52.5)
	2-3	400 (42.1)
	>3	51 (5.4)

Personal history of hypertension	Present	176 (18.5)
	Absent	716 (75.4)
	Do not know	57 (6.0)

Caregiving duration/day	<1 hour	512 (54.0)
	1–3 hours	309 (32.6)
	4-5 hours	65 (6.8)
	5–10 hours	23 (2.4)
	10–15 hours	13 (1.4)
	>15 hours	27 (2.8)

Source of knowledge about hypertension	Doctor	793 (83.6)
	Family/relative/friends/neighbors	536 (56.5)
	Social media	382 (40.4)
	Do not know	14 (1.5)

**Table 2:** KAP scoring.

KAP questions	Scoring (mean ± sd)
Knowledge questions	
(1) A blood pressure level of less than 120/80 mmHg is considered to be high (no) (0/1)	0.555 ± 0.497
(2) Hypertension is a lifelong disease (yes) (0/1)	0.453 ± 0.498
(3) The older patients have higher risk of having hypertension. (yes) (0/1)	0.832 ± 0.374
(4) Men has a higher risk of hypertension compared to women (yes) (0/1)	0.158 ± 0.365
(5) Do you know the danger signs of hypertension? (yes) (0–5)	2.301 ± 1.536
(6) Hypertension is a risk factor for stroke (yes) (0/1)	0.977 ± 0.151
(7) Stroke is a disease of the brain (yes) (0/1)	0.797 ± 0.403
(8) Do you know the name of the medication your patient is on? (yes/no) (0/1)	0.648 ± 0.478
(9) Do you know the side effects of the medication he is on? (dry cough, ankle edema) (yes) (0/1)	0.345 ± 0.475
(10) Hypertension can lead to other life-threatening diseases. (yes) (0/1)	0.610 ± 0.488
**Total knowledge percentage (0–100)**	**54.83** **±** **17.95**

Attitude questions	
(1) I agree that even the pressure remains normal and symptoms release, the medication should be continued. (−2 to +2)	0.734 ± 1.125
(2) I agree that my patient should do regular exercise. (−2 to +2)	1.195 ± 0.895
(3) I agree that my patient should avoid extra salt. (−2 to +2)	1.309 ± 0.919
(4) I agree that my patient should take medication regularly. (−1 to +1)	0.934 ± 0.303
(5) I agree that my patient should have enough consumption of healthy diet (−2 to +2)	0.689 ± 1.100
(6) Hypertension can be controlled with herbal medicine. (−2 to +2)	0.414 ± 0.686
**Total attitude percentage (0–100)**	**47.95** **±** **24.05**

Practice questions	
(1) Buy medicines for the patient regularly (0/1)	0.487 ± 0.500
(2) Help the patient to take medicines regularly (0/1)	0.314 ± 0.464
(3) Remind the patient to take the medicines regularly (0/1)	0.727 ± 0.446
(4) Looking out/being conscious about danger signs like severe chest pain, severe headache, became unconscious, had nasal bleeding, numbness of a side of the body, vomiting, etc. (0/1)	0.906 ± 0.292
(5) Encourage patient to avoid smoking (0/1)	0.366 ± 0.482
(6) Encourage patient to avoid extra salt, red meat, fatty foods, and egg yolk (0/1)	0.804 ± 0.397
(7) Encourage patient to do exercise regularly for 30 minutes for 5 days/week (0/1)	0.652 ± 0.477
(8) Encourage patient to avoid stress (0/1)	0.725 ± 0.447
(9) Encourage patient to take 6–8 hours of sleep daily (0/1)	0.593 ± 0.492
(10) Take the patient for regular medical check-up. (0/1)	0.552 ± 0.498
**Total practice percentage (0–100)**	**61.26** **±** **17.50**

**Table 3:** Correlations between factors and KAP percent score.

Factors	Knowledge	Attitude	Practice
Living with the patient in the same home	*P* = 0.004	*P* = 0.002	*P* < 0.001
Yes	54.95 ± 17.33	48.86 ± 23.72	62.24 ± 16.58
No	45.86 ± 20.31	35.17 ± 30.05	51.58 ± 19.39
Sometimes	56.96 ± 20.52	45.85 ± 22.92	57.74 ± 21.36

Relation with the patient	*P* **<** **0.001**	*P* **<** **0.001**	*P* = 0.304
Spouse	50.18 ± 15.86	44.96 ± 24.23	61.52 ± 15.92
Daughter/Son	58.34 ± 17.37	51.44 ± 22.27	61.92 ± 17.69
Daughter-in-law/Son-in-law	48.45 ± 15.13	46.25 ± 22.50	57.61 ± 16.22
Sister/Brother	53.92 ± 19.42	53.67 ± 25.86	62.90 ± 14.19
Others	50.76 ± 20.54	38.49 ± 27.16	59.29 ± 19.81

Gender	*P* = 0.917	*P* = 0.925	*P* = 0.057
Male	54.90 ± 17.92	47.86 ± 23.91	62.53 ± 18.45
Female	54.78 ± 18.00	48.01 ± 24.17	60.31 ± 16.70

Age of the caregiver (in years)	*P* **<** **0.001**	*P* = 0.119	*P* = 0.363
18–25	57.55 ± 18.30	48.79 ± 22.92	60.88 ± 18.02
26–30	55.58 ± 18.61	50.30 ± 25.78	62.55 ± 17.45
31–35	49.74 ± 17.26	47.75 ± 24.46	59.16 ± 16.47
36–40	47.27 ± 14.25	41.16 ± 26.23	59.27 ± 19.89
>40	51.94 ± 16.45	46.20 ± 24.48	62.77 ± 15.84

Education	*P* **<** **0.001**	*P* **=** **0.001**	*P* **<** **0.001**
Illiterate	39.5 ± 15.18	29.41 ± 31.08	54.12 ± 12.78
Primary	42.96 ± 13.81	41.32 ± 24.51	56.06 ± 17.00
Secondary	47.09 ± 13.45	45.70 ± 22.87	57.61 ± 17.13
Higher secondary	53.49 ± 18.08	49.68 ± 23.19	60.64 ± 18.00
Tertiary	59.68 ± 17.61	49.05 ± 24.11	63.49 ± 17.17

Occupation	*P* **<** **0.001**	*P* **<** **0.001**	*P* = 0.066
Student	54.08 ± 16.69	46.18 ± 23.19	62.48 ± 18.28
Homemaker	47.03 ± 13.86	44.49 ± 25.24	58.78 ± 15.63
Service holder	54.22 ± 16.24	50.00 ± 25.66	62.17 ± 17.69
Business	53.30 ± 16.37	49.36 ± 20.65	64.30 ± 15.35
Health professionals	68.10 ± 17.86	54.40 ± 22.53	60.37 ± 16.87
Others	38.31 ± 16.54	36.64 ± 27.41	57.88 ± 25.83

Residence	*P* **<** **0.001**	*P* **<** **0.001**	*P* **=** **0.001**
Rural	44.72 ± 15.08	42.63 ± 24.71	58.45 ± 18.99
Semiurban	52.53 ± 17.67	45.59 ± 23.25	58.59 ± 17.18
Urban	58.44 ± 17.60	50.25 ± 23.86	62.99 ± 16.98

Personal history of hypertension	*P* **=** **0.001**	*P* **=** **0.019**	*P* **<** **0.001**
Present	53.94 ± 18.24	48.40 ± 25.75	56.42 ± 18.65
Absent	55.72 ± 17.83	48.53 ± 23.02	62.89 ± 16.70
Do not know	46.49 ± 16.54	39.23 ± 29.42	55.79 ± 19.91

Caregiving duration/day	*P* = 0.207	*P* **=** **0.025**	*P* = 0.981
<1 hour	56.18 ± 19.03	48.26 ± 24.20	61.04 ± 17.57
1–3 hours	53.03 ± 16.34	49.54 ± 21.40	61.42 ± 17.26
4-5 hours	53.63 ± 18.26	41.40 ± 25.56	62.00 ± 18.56
5–10 hours	55.90 ± 11.73	38.34 ± 35.94	59.57 ± 17.96
10–15 hours	50.55 ± 18.08	38.46 ± 28.30	63.08 ± 17.02
>15 hours	53.97 ± 17.31	52.19 ± 28.14	62.59 ± 17.50

Duration of caregiving (years)	*P* **=** **0.036**	*P* = 0.065	*P* **=** **0.002**
<1	55.23 ± 20.58	50.11 ± 26.19	57.07 ± 18.56
1–5	54.79 ± 17.68	48.38 ± 23.54	62.13 ± 17.58
5–10	57.07 ± 17.10	49.21 ± 23.51	63.22 ± 16.50
>10	51.28 ± 18.22	42.94 ± 25.09	57.39 ± 17.21

Number of hypertensive people in the family	*P* **=** **0.037**	*P* = 0.364	*P* **=** **0.013**
1	53.53 ± 18.36	47.30 ± 24.54	60.02 ± 17.36
2-3	55.96 ± 17.08	48.20 ± 23.41	63.18 ± 17.59
>3	58.68 ± 19.81	52.23 ± 24.11	58.43 ± 17.01

**Table 4:** Correlations between factors and burdens.

Variables	Effect on mood/activities					Tiredness					Physical restlessness/tremor/stiffness					Financial burden				
	0	1	2	3	4	0	1	2	3	4	0	1	2	3	4	0	1	2	3	4
Sharing same home	*P* **=** **0.026**					*P* **=** **0.049**					*P* **=** **0.005**					*P* = 0.196				
Yes	51.9	21.4	12.2	10.1	4.5	58.9	17.6	12.3	7.7	3.5	60.9	18.6	11.8	5.4	3.3	55.7	17.5	10.4	9.3	7.2
No	44.7	15.8	13.2	23.7	2.6	47.4	13.2	10.5	18.4	10.5	36.8	21.1	15.8	18.4	7.9	42.1	18.4	10.5	23.7	5.3
Sometimes	47.8	32.2	9.6	5.2	5.2	61.7	20.9	6.1	7.0	4.3	56.5	24.3	8.7	4.3	6.1	51.3	22.6	9.6	8.7	7.8

Relation with the patient	*P* **<** **0.001**					*P* **<** **0.001**					*P* **<** **0.001**					*P* **<** **0.001**				
Spouse	44.6	24.5	14.2	11.8	4.9	48.5	20.6	21.6	6.9	2.5	58.3	22.5	13.7	2.0	3.4	51.5	13.7	9.8	11.8	13.2
Daughter/Son	59.4	21.1	8.5	7.4	3.6	69.1	15.7	5.9	6.3	3.0	65.8	17.8	9.1	4.6	2.7	59.6	19.5	8.5	7.8	4.6
Daughter-in-law/Son-in-law	21.7	21.7	26.1	17.4	13.0	26.1	17.4	19.6	19.6	17.4	34.8	15.2	17.4	17.4	15.2	23.9	21.7	19.6	17.4	17.4
Sister/brother	45.2	32.3	9.7	9.7	3.2	58.1	25.8	6.5	6.5	3.2	48.4	29.0	6.5	16.1	0.0	48.4	25.8	9.7	12.9	3.2
Others	40.4	22.7	17.0	14.9	5.0	46.1	19.9	16.3	12.8	5.0	47.5	19.9	17.0	9.9	5.7	51.8	16.3	14.9	11.3	5.7

Gender	*P* = 0.424					*P* **=** **0.003**					*P* = 0.195					*P* **=** **0.020**				
Male	52.7	23.3	11.5	9.3	3.2	65.4	16.9	8.6	6.1	2.9	61.0	18.9	11.3	6.6	2.2	56.6	21.1	8.6	7.1	6.6
Female	49.9	21.8	12.2	10.5	5.5	53.8	18.5	13.7	9.4	4.6	58.2	19.8	11.8	5.2	5.0	53.0	15.9	11.6	11.8	7.6

Age of the caregiver (in years)	*P* **=** **0.006**					*P* **<** **0.001**					*P* **=** **0.001**					*P* **<** **0.001**				
18–25	55.9	24.0	9.8	7.7	2.5	64.7	18.2	9.2	6.5	1.5	61.8	20.9	10.9	5.0	1.5	59.3	18.8	10.0	9.2	2.7
26–30	46.0	18.2	13.1	15.3	7.3	59.1	12.4	7.3	16.1	5.1	56.2	16.8	9.5	10.9	6.6	54.7	18.2	8.0	8.0	10.9
31–35	49.4	20.5	9.6	10.8	9.6	60.2	13.3	10.8	7.2	8.4	65.1	14.5	7.2	6.0	7.2	47.0	22.9	13.3	10.8	6.0
36–40	50.9	14.5	16.4	10.9	7.3	49.1	20.0	12.7	3.6	14.5	49.1	18.2	14.5	7.3	10.9	38.2	14.5	12.7	16.4	18.2
>40	43.6	24.6	15.9	11.3	4.6	46.2	22.1	20.0	7.7	4.1	56.4	20.0	15.9	3.6	4.1	50.8	15.4	10.8	10.3	12.8

Education	*P* **<** **0.001**					*P* **<** **0.001**					*P* **<** **0.001**					*P* **<** **0.001**				
Illiterate	17.6	29.4	23.5	17.6	11.8	17.6	29.4	29.4	11.8	11.8	35.3	29.4	11.8	11.8	11.8	23.5	23.5	23.5	11.8	17.6
Primary	34.8	21.2	16.7	15.2	12.1	40.9	15.2	16.7	12.1	15.2	47.0	18.2	13.6	6.1	15.2	31.8	18.2	13.6	16.7	19.7
Secondary	31.0	17.7	19.5	19.5	12.4	37.2	15.0	23.0	19.5	5.3	36.3	19.5	21.2	14.2	8.8	29.2	20.4	19.5	11.5	19.5
Higher secondary	53.2	23.9	11.1	10.0	1.8	57.5	23.2	10.0	6.8	2.5	62.1	22.1	9.6	4.3	1.8	58.2	20.7	7.1	8.9	5.0
Tertiary	58.1	22.6	9.5	6.8	3.0	68.7	15.2	8.2	5.3	2.5	66.0	17.5	10.1	4.4	1.9	62.8	15.9	9.1	8.9	3.4

Occupation	*P* **<** **0.001**					*P* **<** **0.001**					*P* **<** **0.001**					*P* **<** **0.001**				
Student	53.0	24.8	11.4	7.9	2.9	62.2	18.1	10.2	7.3	2.2	61.3	20.6	12.4	4.4	1.3	56.8	20.3	10.8	9.5	2.5
Homemaker	27.9	23.9	17.3	19.3	11.7	32.5	20.3	19.8	17.3	10.2	42.6	21.8	16.2	8.1	11.2	36.0	17.3	13.7	15.2	17.8
Service holder	64.2	20.0	9.2	4.2	2.5	69.2	16.7	9.2	2.5	2.5	66.7	16.7	10.0	5.0	1.7	61.7	17.5	10.0	6.7	4.2
Business	51.6	21.5	15.1	10.8	1.1	59.1	18.3	15.1	6.5	1.1	61.3	17.2	11.8	8.6	1.1	45.2	21.5	10.8	9.7	12.9
Health professionals	67.0	18.8	6.3	5.8	2.1	77.0	14.1	4.2	2.6	2.1	70.7	17.3	6.3	2.6	3.1	72.3	14.7	5.8	5.2	2.1
Others	30.3	24.2	18.2	18.2	9.1	39.4	24.2	15.2	15.2	6.1	45.5	21.2	12.1	18.2	3.0	42.4	15.2	12.1	18.2	12.1

Residence	*P* **<** **0.001**					*P* **<** **0.001**					*P* **<** **0.001**					*P* **<** **0.001**				
Rural	30.4	17.4	21.7	21.1	9.3	37.9	15.5	21.7	17.4	7.5	38.5	17.4	18.6	17.4	8.1	30.4	15.5	18.0	18.6	17.4
Semiurban	45.6	28.2	12.1	7.8	6.3	51.9	23.3	12.6	7.3	4.9	52.4	27.2	11.2	3.4	5.8	51.0	20.4	10.2	8.7	9.7
Urban	58.8	21.8	9.1	7.7	2.6	67.0	16.5	8.2	5.7	2.6	67.7	17.2	9.8	3.4	1.9	62.5	18.0	8.2	7.7	3.4

Number of hypertensive people in the family	*P* = 0.187					*P* = 0.480					*P* = 0.324					*P* = 0.759				
1	51.6	22.3	10.8	12.0	3.2	61.0	15.5	12.0	8.0	3.4	62.2	17.3	10.4	6.8	3.2	56.4	16.9	9.2	9.8	7.6
2-3	49.8	23.3	13.5	7.5	6.0	56.8	20.8	10.5	8.0	4.0	55.8	22.0	12.8	4.8	4.8	51.8	19.5	11.5	10.3	7.0
>3	56.9	17.6	9.8	9.8	5.9	52.9	17.6	13.7	7.8	7.8	60.8	19.6	13.7	3.9	2.0	58.8	19.6	11.8	5.9	3.9

Personal history of hypertension	*P* **=** **0.003**					*P* **=** **0.001**					*P* **<** **0.001**					*P* = 0.062				
Present	47.7	22.2	14.8	8.0	7.4	51.1	20.5	15.3	7.4	5.7	49.4	24.4	15.3	5.7	5.1	49.4	20.5	13.1	8.5	8.5
Absent	53.2	22.6	10.1	10.5	3.6	62.6	16.5	9.8	8.1	3.1	63.8	17.6	10.3	5.0	3.2	57.4	16.9	9.4	9.8	6.6
Do not know	35.1	21.1	26.3	10.5	7.0	35.1	26.3	21.1	8.8	8.8	35.1	26.3	15.8	15.8	7.0	35.1	26.3	14.0	14.0	10.5

Caregiving duration/day	*P* **<** **0.001**					*P* **<** **0.001**					*P* **<** **0.001**					*P* **<** **0.001**				
<1 hour	63.7	22.5	7.2	5.3	1.4	72.1	17.6	5.5	4.5	0.4	69.3	18.9	7.4	3.3	1.0	64.6	17.6	8.0	6.8	2.9
1–3 hours	38.8	23.9	16.8	16.8	3.6	48.2	17.8	18.8	12.3	2.9	50.2	21.0	17.5	8.4	2.9	44.0	20.1	12.3	13.6	10.0
4-5 hours	15.4	23.1	21.5	20.0	20.0	21.5	18.5	23.1	16.9	20.0	32.3	21.5	15.4	12.3	18.5	21.5	21.5	20.0	13.8	23.1
5–10 hours	34.8	13.0	21.7	4.3	26.1	26.1	26.1	13.0	4.3	30.4	43.5	17.4	13.0	4.3	21.7	47.8	13.0	4.3	17.4	17.4
10–15 hours	30.8	23.1	15.4	7.7	23.1	23.1	23.1	30.8	0.0	23.1	30.8	23.1	15.4	15.4	15.4	46.2	7.7	30.8	7.7	7.7
>15 hours	63.0	11.1	11.1	3.7	11.1	63.0	11.1	3.7	11.1	11.1	70.4	3.7	11.1	3.7	11.1	74.1	7.4	3.7	7.4	7.4

Duration of caregiving (years)	*P* = 0.355					*P* **=** **0.001**					*P* = 0.289					*P* = 0.742				
<1	58.5	18.3	8.5	8.5	6.1	59.8	19.5	4.9	4.9	11.0	67.1	17.1	7.3	3.7	4.9	62.2	18.3	8.5	6.1	4.9
1–5	52.6	23.2	11.4	9.1	3.6	62.3	17.1	9.7	7.8	3.0	61.3	18.5	10.5	6.3	3.4	55.8	18.5	9.5	9.3	6.9
5–10	49.5	21.2	13.0	9.6	6.7	58.7	16.8	13.0	8.2	3.4	56.3	21.6	11.5	6.7	3.8	51.9	17.3	13.0	10.1	7.7
>10	43.3	23.9	14.2	14.9	3.7	44.8	20.9	20.1	10.4	3.7	52.2	20.9	18.7	3.7	4.5	49.3	17.9	10.4	13.4	9.0

Bold values represent the significant *P* values.

## Data Availability

The data used for this project are available upon reasonable request to the corresponding author.

## Additional Points

Although there are several strengths in our study as we investigated the correlations of KAP and burden with the caregivers' information, there are some limitations too. The association of KAP and burden with patient information should be distinguished in future studies.
